# P-539. Does Switching from Triple (or Quadruple)-Drug Regimen to Double-Drug Regimen with Oral INSTIs Reduce Drug-Related Adverse Events (DRAEs) and Toxicity in Virologically Suppressed People with HIV? SATISFACTION Study

**DOI:** 10.1093/ofid/ofae631.738

**Published:** 2025-01-29

**Authors:** Antonio Antela, Pere Domingo-Pedrol, Francisco Mariano Jover-Díaz, Javier Martínez-Sanz

**Affiliations:** Hospital Clínico Universitario de Santiago de Compostela, Santiago de Compostela, Galicia, Spain; Hospital Universitario de la Santa Creu i Sant Pau, Barcelona, Catalonia, Spain; Hospital Clínico Universitario de San Juan, Alicante, Comunidad Valenciana, Spain; Hospital Ramón y Cajal, Madrid, Madrid, Spain

## Abstract

**Background:**

Triple or quadruple drug regimens (3 or 4DR) remain the gold standard for HIV treatment. However, there is currently a trend towards therapy simplification from 3 or 4DR to double-drug regimens (2DR) based on Integrase Strand Transfer Inhibitors (INSTIs). Switching to 2DR is not based on increased efficacy, as studies suggest non-inferiority of 2DR, but aims to reduce adverse events and long-term toxicity associated with prolonged exposure to current antiretroviral drugs. However, to date, neither clinical trials nor real-world data have confirmed this hypothesis.

This study aims to describe the proportion of DRAEs reported in phase III and IV studies in virologically suppressed people with HIV (PWH) on 3 or 4DR and those who have switched from 3 or 4DR to 2DR with oral INSTIs at ≥ 48 weeks.

Percentage of DRAEs and DRAEs leading to discontinuation

**Figure:**
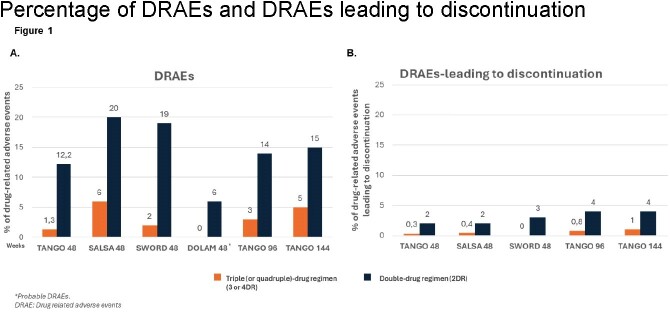

Percentage of DRAEs and DRAEs leading to discontinuation

**Methods:**

A systematic literature review was conducted searching on the main databases (PubMed, ClinicalTrials.gov, EU Clinical Trials Register, Cochrane and Embase) from March 2014 to March 2024, as well as on the main conferences from March 2022 to March 2024. We included phase III and phase IV studies evaluating the switch from 3 or 4DR to 2DR based on oral second-generation INSTIs in virologically suppressed PWH, with ≥ 48 weeks of follow-up. A meta-analysis is being conducted in studies with 48-week follow-up. Financial support: Gilead Sciences

**Results:**

Nine publications met the inclusion criteria. Eight publications corresponded to three phase III clinical trials (TANGO, SALSA and SWORD) at different follow-up periods and one to a phase IV study (DOLAM), with a 48-week follow-up.

The percentage of DRAEs and DRAEs leading to discontinuation increased in patients who switched to 2DR ([6-20%] and [2-4%], respectively) compared to those who remained in 3 or 4DR ([0-6%] and [0-1%]) at 48 weeks in all studies (Figure 1). A similar trend was observed with longer follow-up times. Differences observed in laboratory parameters when switching from 3 or 4DR to 2DR were not clinically relevant.

**Conclusion:**

Therapy simplification from 3 or 4DR to 2DR with oral second-generation INSTIs in virologically suppressed PWH at ≥ 48 week did not enhance the safety and tolerability profile compared with 3 or 4DR continuation, according to data reported in phase III clinical trials and phase IV studies.

**Disclosures:**

**Antonio Antela, MD, PhD**, Gilead: Honoraria|Janssen: Advisor/Consultant|MSD: Honoraria|ViiV: Board Member **Pere Domingo-Pedrol, Medical Doctor**, Gilead: Advisor/Consultant|Gilead: Honoraria|Janssen: Advisor/Consultant|Janssen: Honoraria|MSD: Advisor/Consultant|MSD: Honoraria|ViiV: Advisor/Consultant|ViiV: Honoraria **Francisco Mariano Jover-Díaz, Medical Doctor**, Gilead: Advisor/Consultant|Gilead: Honoraria|Janssen: Advisor/Consultant|ViiV: Advisor/Consultant **Javier Martínez-Sanz, Medical Doctor**, Gilead Science: Advisor/Consultant|Gilead Science: Grant/Research Support|Gilead Science: Honoraria|Janssen: Advisor/Consultant|Janssen: Honoraria|ViV: Honoraria

